# Plasma proteomic profile of inflammatory depressive symptoms

**DOI:** 10.1016/j.bbih.2026.101311

**Published:** 2026-07-23

**Authors:** Filip Ängeby, Filip Ventorp, Miranda Stiernborg, Klara Suneson, Gustav Söderberg Veibäck, Jesper Lindahl, Johanna Tjernberg, Darya Ståhl, Jonas Berge, Catharina Lavebratt, Daniel Lindqvist

**Affiliations:** a Unit for Biological and Precision Psychiatry, Department of Clinical Sciences Lund, Lund University, Lund, Sweden; b Department of Psychiatry, Skåne University Hospital, Lund, Sweden; c Department of Psychiatry, Skåne University Hospital, Malmö, Sweden; d Department of Gastroenterology and Nutrition, Skåne University Hospital, Malmö, Sweden; e Department of Clinical Sciences Lund, Psychiatry, Lund University, Lund, Sweden; f Addiction Center, Malmö, Sweden; g Department of Molecular Medicine and Surgery, Karolinska Institutet, and Center for Molecular Medicine, Karolinska University Hospital, Stockholm, Sweden; h Infectious Disease and Microbiome Program, Broad Institute, Cambridge, MA, USA

## Abstract

**Background:**

Previous studies suggest that specific depressive symptoms such as low energy, fatigue, sleep and appetite disturbances are associated with systemic low-grade inflammation. In this study, we investigated associations between an inflammatory symptom profile of depression and proteomic markers spanning inflammatory and metabolic pathways.

**Methods:**

We analyzed 158 proteins related to inflammation and metabolism using proximity extension assay in blood samples from 165 patients with Major Depressive Disorder (MDD). Severity of inflammatory depressive symptoms was assessed using a composite score summarizing Patient Health Questionnaire-9 (PHQ-9) items related to fatigue and sleep/appetite disturbances. We used partial least squares (PLS), PLS discriminant analysis (PLS-DA) and linear regression models to identify proteins related to inflammatory depressive symptom severity.

**Results:**

Out of the 158 proteins, 14 were selected by either PLS or PLS-DA. After false discovery rate correction, proteins identified by PLS or PLS-DA that remained significantly associated with more severe inflammatory depressive symptoms included several immunometabolic biomarkers, namelyinterleukin-6 (IL-6), hepatocyte growth factor, oncostatin M, thrombospondin-4, low affinity immunoglobulin gamma Fc region receptor II-a and intercellular adhesion molecule 1. With the exception of IL-6, none of these markers showed significant associations with the remaining PHQ-9 items that were not classified as inflammatory depressive symptoms.

**Conclusions:**

These findings support the growing body of evidence linking inflammatory and metabolic protein alterations to specific depressive symptoms.

## Highlights

• Inflammation is known to be selectively associated with specific depressive symptoms.
• Advances in proteomics allow simultaneous assays of large numbers of proteins.
• We found links between immunometabolic markers and specific depressive symptoms.
• We highlight depression heterogeneity and possible differences in pathophysiology.

## 1 Introduction

Patients with major depressive disorder (MDD) have higher mean levels of blood inflammatory markers compared to non-MDD controls (Haapakoski et al., 2015). Several studies suggest that low-grade inflammation is more common in an MDD subgroup with certain depressive symptoms such as sleep disturbance, fatigue and abnormal appetite (Frank et al., 2021; Fried et al., 2020; Jokela et al., 2016; Suneson et al., 2023). This has led to the hypothesis that, in a subgroup of patients with MDD, chronic low-grade inflammation may contribute to the development of depressive symptoms – a proposed subtype often referred to as “inflammatory depression”. Evidence suggests that this profile may characterize approximately one-third of patients with MDD (Osimo et al., 2019). Potential treatments targeting inflammatory depressive symptoms include both non-pharmacological interventions, such as dietary treatments and exercise, as well as anti-inflammatory medications (Penninx et al., 2025; Suneson et al., 2021). Although several studies suggest that patients with more pronounced inflammatory depressive symptoms and elevated inflammatory markers may have a preferential effect of such treatments, the current evidence is insufficiently robust to support precision treatment of anti-inflammatory interventions in MDD (Penninx et al., 2025). Improved understanding of the biological processes underlying inflammatory depression would allow for better enrichment strategies and the selection of outcome measures more closely aligned with these mechanisms in future treatment studies, which could advance precision psychiatry.

So far, most studies investigating biomarkers of depressive symptoms tentatively linked to inflammation have typically assayed a limited number of proteins such as C-reactive protein (CRP), tumor necrosis factor-alpha (TNF-α) and interleukin-6 (IL-6). Employing a broader proteomic approach has been proposed as a means to gain greater mechanistic insights and identify new biomarkers of the inflammatory depression subtype (Milaneschi et al., 2020; Penninx et al., 2025). Assuming homogeneity in complex and multifactorial psychiatric disorders such as MDD can be detrimental for research that strives to understand underlying pathophysiological mechanisms (Feczko and Fair, 2020). Proteomic studies have not found any disease-specific pathways, and using larger and more well-defined clinical samples (*e.g.* investigating specific symptom profiles) has been proposed as a way to detect separation between phenotypes (Comes et al., 2018).

In this study, we investigated the relationship between inflammatory depressive symptoms and a broad panel of immunometabolic biomarkers assessed using proteomics.

## 2 Methods

### 2.1 Clinical data

Clinical data was collected from 176 MDD patients during baseline visits in two antidepressant treatment studies conducted at the psychiatric research unit in Lund, Sweden between 2017 and 2023. These trials investigated the adjunctive antidepressant efficacy of omega-3 fatty acids and probiotics respectively, and the main findings have been published elsewhere (Soderberg Veiback et al., 2025; Suneson et al., 2024). Both studies were pre-registered at clinicaltrials.gov (ref#NCT03660280 and ref#NCT03143075) and received ethical approval by the ethical review board in Lund, Sweden. The studies included patients with MDD but differed slightly in inclusion and exclusion criteria. For instance, the Probiotics sample was enriched for low-grade inflammation using high-sensitive C-reactive protein (hs-CRP) ≥ 1 mg/L and overweight (body mass index (BMI) ≥ 25 kg/m^2^), whereas the Omega-3 sample included MDD patients regardless of hs-CRP levels or BMI. Several exclusion criteria were the same in both studies including high suicide risk, co-morbid psychotic or bipolar disorder, pregnancy, substance use disorder and ongoing infection. In order to exclude patients with infection, hs-CRP was quantified at the study visit, subjects were asked about infectious symptoms, and underwent physical examinations. Inflammatory depressive symptoms were assessed using the patient health questionnaire-9 (PHQ-9) (Kroenke et al., 2001), where items #3 (trouble falling or staying asleep, or sleeping too much), #4 (feeling tired or having little energy), and #5 (poor appetite or overeating) were extracted to create a composite Inflammatory Depressive Symptoms (InfDep) Score (range 0-9). These specific PHQ-9 items were pre-selected since they had been found to be associated with low-grade inflammation in a previous meta-analysis (Jokela et al., 2016), and was pre-registered as a secondary outcome measure. Similar items from other depression rating scales have also been found to be associated with inflammatory biomarkers in subsequent studies (Frank et al., 2021; Fried et al., 2020; White et al., 2017).

### 2.2 Blood sampling and protein assays

EDTA plasma from venipuncture was taken at the baseline visits after fasting and stored in a biobank at −80 °C, until analyzed using proximity extension assay (PEA), which is a method for multiplex protein measurement described in detail elsewhere (Assarsson et al., 2014). Briefly, PEA measures the product of a polymerase chain reaction that is initiated when a single-stranded oligonucleotide coupled to an antibody binds to a target protein simultaneously with a matching single-stranded antibody-coupled oligonucleotide. We selected two Olink protein panels – Target96 Cardiometabolic (“the cardiometabolic panel”) and Target96 Inflammation (“the inflammation panel”) which were analyzed at the certified service provider Affinity Proteomics (SciLifeLab, Uppsala, Sweden) and in total measured 184 protein markers. Nine 96-well plates were run for each Olink panel including singlet plasma samples from all 176 patients, with 8 technical controls and 88 patient samples (spiked with four internal controls) on each plate. In subsequent analyses we included only samples that were above the lower limit of detection (LOD) in 90 % of the patients, rendering 72 proteins in the inflammation panel and 86 in the cardiometabolic panel. Internal quality control resulted in data from 157 patients in the cardiometabolic panel and 165 patients in the inflammation panel to be further assessed. Normalized expression unit (NPX) values – the output of a PEA – are relative. To enable comparison of results from both studies, the plate data were bridged using duplicate samples from healthy controls (not analyzed in this study, n = 49) included in each PEA, and normalization was performed as specified in R package OlinkAnalyze (https://cran.r-project.org/web/packages/OlinkAnalyze/index.html, v4.3.2.).

### 2.3 Statistical analyses

To identify a limited number of proteins tentatively associated with inflammatory depressive symptoms, partial least squares discriminatory analysis (PLS-DA) was performed using R-package mixOmics (http://mixOmics.org, v6.32.0). PLS-DA reduces dimensionality in large datasets by creating “latent components”, which are loaded with a subset of variables (in this case a subset of the proteins) that explain the most co-variance between pre-defined groups (Le Cao et al., 2011). “High” and “low” InfDep groups were created by splitting the sample at the median of the InfDep score. As an illustrative analogy, we regard the latent component created by PLS-DA (from here referred to simply as “component”) an indirect measure or proteomic “fingerprint” of the depressive symptoms inquired with the InfDep score. The PLS-DA protocol was almost identical to our previous publication (Lindahl et al., 2025) but differed slightly because of a larger sample size in the current study, allowing us to perform 22-fold cross-validations repeated 100 times, and using a proportionally smaller “test set” (on which the model's prediction accuracy was evaluated) consisting of 20 % of all subjects. The number of PLS-DA components (in the remaining 80 % "training set") was determined by minimizing the cross-validated classification error using the max. dist distance rule. If the model could not automatically choose a number of components, one component (which is total number of classes minus one) was chosen as advised by mixOmics authors (Le Cao et al., 2011). This was the case in the PLS-DA on the inflammation panel, where one component containing eight proteins was chosen. In the cardiometabolic panel, the PLS-DA model initially selected two components containing almost all proteins and thus did not fulfill its purpose as a feature selector. PLS-DA is known to be prone to over-fitting, so to restrict the number of proteins and only identify key biomarkers we used a stability score cut-off of 0.8, meaning only proteins that were identified in ≥80 % of cross-validation folds were included in the final output. On the cardiometabolic panel where almost all proteins were initially included (indicating over-fitting), we made further adjustments by setting the maximum number of proteins to include in a component to 20, which resulted in a significant reduction of one component containing three proteins in the final output. Internal testing of this component showed lower area under the receiver operating characteristic (AUROC), but better prediction accuracy on the test set (0.533 vs 0.467, see supplementary material 1) which indicates greater generalizability. The component chosen by PLS-DA from the inflammatory panel had even better prediction accuracy than the cardiometabolic panel (0.625 vs 0.533), comparable to our previous publication where PLS-DA was used to discriminate between MDD patients and controls (Lindahl et al., 2025).

As a complementary statistical approach, to validate findings from PLS-DA and explore if linear associations on the ten-point InfDep scale and protein levels could be identified by not performing an arbitrary division of the study population by the median, we performed partial least squares (PLS) regression (Abdi and Williams, 2013) which is also included in the mixOmics R-package. The PLS was set to *regression mode*, creating (latent) components based on the linear and asymmetrical relationship between the protein markers and continuous InfDep scores. InfDep values were normalized by Blom-transformation using R-package rcompanion (https://CRAN.R-project.org/package=rcompanion, v2.5.0) prior to PLS analysis, as advised by mixOmics. All cross-validations in the PLS were done 26-fold and repeated 100 times. The number of components was decided by the algorithm using cross-validation, where the value of adding a dimension was assessed using the $Q^{2}$ criterion. Next, the number of proteins to be retained in each component was decided based on the mean absolute error. Performance of the final PLS model was evaluated by correlating the selected component and Blom-transformed InfDep score, and by assessing the mean squared error prediction. Identifying proteins based on continuous InfDep scores with PLS resulted in one component containing five proteins in both the inflammatory and cardiometabolic panel. Complete performance metrics for both PLS and PLS-DA can be found in the supplementary material 1, and a list of all proteins selected can be found in Table 2.

To test associations between symptoms and protein levels in our dataset, the protein markers selected by PLS and PLS-DA were analyzed using linear regression with NPX values for each protein as dependent variable and either Blom-transformed InfDep scores (for proteins chosen by PLS) or InfDep groups based on the median split (for proteins chosen by PLS-DA) as the independent variable. If NPX values for a marker were deemed non-normally distributed by the Shapiro-Wilk test p-vales generated by the corresponding regression were calculated by permutation using the R package lmPerm (https://cran.r-project.org/web/packages/lmPerm/index.html, v2.1.4). Adjustments were made for age and sex, but not for BMI since we considered overweight a part of the inflammatory depression subtype that we did not want to factor out (Lasserre et al., 2014; Luppino et al., 2010; Wijaya et al., 2022), consistent with previous publicationsusing a similar approach as ours (Lamers et al., 2016; van Haeringen et al., 2023). False discovery rate (FDR)-corrections were made according to Benjamini-Hochberg (Benjamini and Hochberg, 2018).

### 2.4 STRING network analysis

To investigate whether the identified proteins were involved in shared biological processes, network analysis was performed using STRING database v 12.0 (Szklarczyk et al., 2023). The STRING database collects, integrates and quantifies protein-protein interactions systematically using data from several sources (automated text mining from scientific literature, computational interaction predictions from co-expression, conserved genomic context, databases of interaction experiments and known complexes or pathways from curated sources) creating a “interaction score” for each association ranging from 0 to 1, which corresponds to the estimated likelihood of a given association being true given the underlying evidence.

## 3 Results

### 3.1 Demographic characteristics

Descriptive characteristics in the high and low InfDep score groups are summarized in Table 1. Most subjects were female, with the same proportion between-groups. As expected, the high InfDep group had higher BMI and higher hs-CRP compared to the low InfDep group.

**Table 1:** Descriptive statistics on the study population divided by median split Inflammatory Depressive Symptom (InfDep) group. All continuous values are mean (SD) except for hs-CRP which is shown as median [min, max]. Categorical values are given as n (%).

Characteristic	High InfDep group n = 70	Low InfDep group n = 106	*P*-value
BMI (kg/m^2^)	31 (7)	27 (6)	<0.001 ^c^
Sex			>0.999 ^d^
Male	15 (21 %)	22 (21 %)	
Female	55 (79 %)	84 (79 %)	
Age (years)	40 (13)	42 (13)	0.429^c^
Smoking status			0.293 ^e^
Never	60 (87 %)	83 (79 %)	
Sometimes	4 (6 %)	14 (13 %)	
Daily	5 (7 %)	8 (8 %)	
Snus use			0.549 ^e^
Never	60 (88 %)	90 (87 %)	
Sometimes	0 (0 %)	3 (3 %)	
Daily	8 (12 %)	11 (11 %)	
HAM-D score ^*a*^	20 (4)	19 (3)	0.106^c^
MADRS score ^*a*^	26 (6)	22 (4)	0.008^c^
CGI-S score			0.776^e^
3 – Mildly ill	16 (48 %)	29 (43 %)	
4 – Moderately ill	17 (52 %)	36 (54 %)	
5 – Markedly ill	0 (0 %)	2 (3 %)	
Previous ECT	1 (2 %)	4 (4 %)	0.649 ^e^
Previous suicide attempt	14 (21 %)	13 (13 %)	0.230 ^d^
hs-CRP (mg/L)	2.9 [0.3, 21.0]	1.7 [0.3, 46.0]	0.006^c^
Study participation			0.077 ^d^
Omega-3	34 (49 %)	67 (63 %)	
Probiotics	36 (51 %)	39 (37 %)	

^b^ hs-CRP values are presented according to the median since levels below 0.6 mg/L could not be detected (<0.6). For group comparisons, these values were set to 0.3 mg/L.

Abbreviations: BMI = body mass index, HAM-D = Hamilton depression rating scale, MADRS = Montgomery-Åsberg Depression rating scale, CGI-S = clinical global impression - severity scale, ECT = electroconvulsive therapy, hs-CRP = high sensitive C-reactive protein.

^a^ Depressive symptoms were assessed using HAM-D interview in the Omega-3 study and MADRS interview in the Probiotics study.

^c^ Wilcoxon signed rank test.

^d^ Chi-square test.

^e^ Fisher's exact test.

**Table 2:** Biomarkers associated with severity of inflammatory depressive symptoms.

Protein marker	Protein panel	Feature selector	Regression coefficient	Non-adjusted P-value	FDR-corrected P-value
IL6	Inflammation	PLS-DA	0.22	0.001	0.006
	Inflammation	PLS	0.19	0.012	0.031
CCL4	Inflammation	PLS-DA	0.14	0.002	0.006
	Inflammation	PLS	0.10	0.059	0.059
HGF	Inflammation	PLS-DA	0.10	0.006	0.015
	Inflammation	PLS	0.08	0.027	0.037
OSM	Inflammation	PLS-DA	0.32	0.026	0.035
	Inflammation	PLS	0.17	0.029	0.037
CCL25	Inflammation	PLS-DA	−0.04	0.620	0.620
TRANCE	Inflammation	PLS-DA	0.16	0.140	0.160
CCL3	Inflammation	PLS-DA	0.10	0.021	0.035
TRAIL	Inflammation	PLS-DA	0.06	0.026	0.035
Flt3L	Inflammation	PLS	0.09	0.008	0.031
THBS4	Cardiometabolic	PLS-DA	0.10	0.013	0.024
	Cardiometabolic	PLS	0.10	0.024	0.050
FCGR2A	Cardiometabolic	PLS-DA	0.13	0.020	0.024
	Cardiometabolic	PLS	0.13	0.030	0.050
ICAM1	Cardiometabolic	PLS-DA	0.14	0.024	0.024
	Cardiometabolic	PLS	0.08	0.018	0.050
FCN2	Cardiometabolic	PLS	0.08	0.086	0.108
UMOD	Cardiometabolic	PLS	0.04	0.122	0.122

Only proteins selected either by partial least squares (PLS) or PLS discriminant analysis (PLS-DA) in relation to Inflammatory Depressive Symptom (InfDep) scores were analyzed with linear regression. The two methods differed where PLS-DA used InfDep groups divided by the median and PLS used continuous, Blom-transformed InfDep scores. The proteins analyzed with linear regression (using the same dichotomized or continuous InfDep scores) were adjusted for age and sex and FDR-corrected. Abbreviations: interleukin-6 (IL-6), C-C motif ligand 4 (CCL4), hepatocyte growth factor (HGF), oncostatin M (OSM), C-C motif ligand 25 (CCL25), TNF-related activation-induced cytokine (TRANCE), C-C motif ligand 3 (CCL3), TNF-related apoptosis-inducing ligand (TRAIL), fms-related tyrosine kinase 3 ligand (Flt3L), thrombospondin-4 (THBS4), low affinity immunoglobulin gamma Fc region receptor II-a (FCGR2A), intercellular adhesion molecule 1 (ICAM1), ficolin-2 (FCN2) and uromodulin (UMOD).

Neither age, clinical global impression severity (CGI-S) scores, proportions of prior electroconvulsive treatment or suicide attempts, nor smoking status or snus (a Swedish moist oral tobacco product) use differed significantly between the high and low InfDep groups.

### 3.2 Associations between inflammatory depressive symptoms and protein markers

Linear regression with FDR-correction on the eight inflammation panel proteins selected by PLS-DA showed that the high InfDep group had significantly higher levels in six of these proteins: interleukin-6 (IL-6), C-C motif ligand 3 (CCL3), C-C motif ligand 4 (CCL4), hepatocyte growth factor (HGF), oncostatin M (OSM) and TNF-related apoptosis-inducing ligand (TRAIL). Out of these, IL-6, OSM and HGF were also selected by PLS showing significant linear relationships with higher InfDep scores. Fms-related tyrosine kinase 3 ligand (Flt3L) was also significant in the linear regression using continuous InfDep scores, though it was not selected by PLS-DA and thus not analyzed with linear regression using the group division. Among the three proteins selected by PLS-DA in the cardiometabolic panel, linear regression showed significantly higher protein levels in the high InfDep group for all: thrombospondin-4 (THBS4), low affinity immunoglobulin gamma Fc region receptor II-a (FCGR2A) and intercellular adhesion molecule 1 (ICAM1) after FDR-correction. These proteins were also selected by PLS, and they had significant linear relationships with increasing InfDep scores after FDR-correction. Results of all regressions are listed in Table 2. A scatterplot showing protein levels in relation to InfDep score of the six proteins that were significant in linear regressions using both dichotomized and continuous InfDep scores can be found in Fig. 2.

**Fig. 1:**
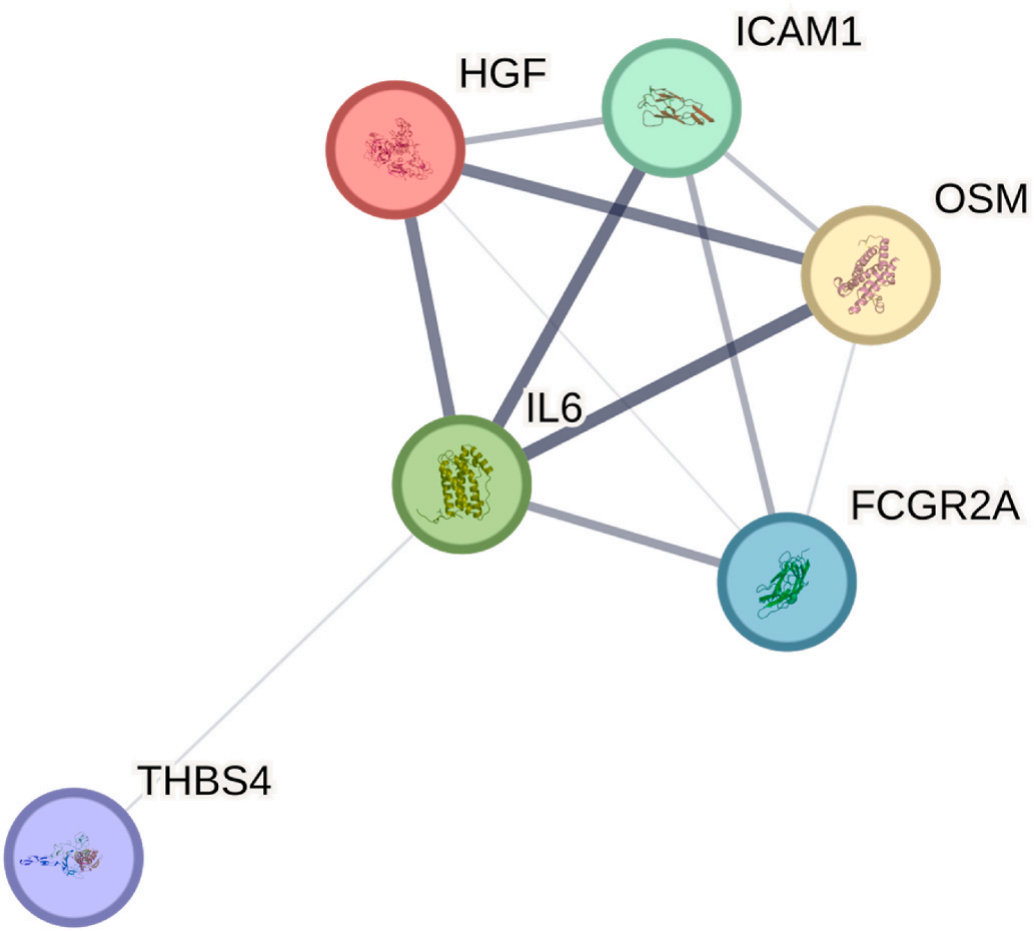
STRING database network associations. Network associations between the proteins that were significantly associated with InfDep scores in linear regressions using both continuous and dichotomized InfDep scores. The lines between proteins are “interaction scores” from the STRING database, where a stronger line indicates increased evidence of a functional association.

Functional associations between proteins were investigated using the STRING database (Fig. 1). Stronger functional association between proteins is visualized with a thicker line, and a minimum required interaction score was set to 0.15 (low confidence). The strongest links were found between HGF/OSM (0.86), OSM/IL-6 (0.96), IL-6/ICAM1 (0.95) and IL-6/HGF (0.86). All proteins but THBS4 had interaction scores ≥0.15 with each other, whereas THBS4 only showed a weak association with IL-6.

**Fig. 2:**
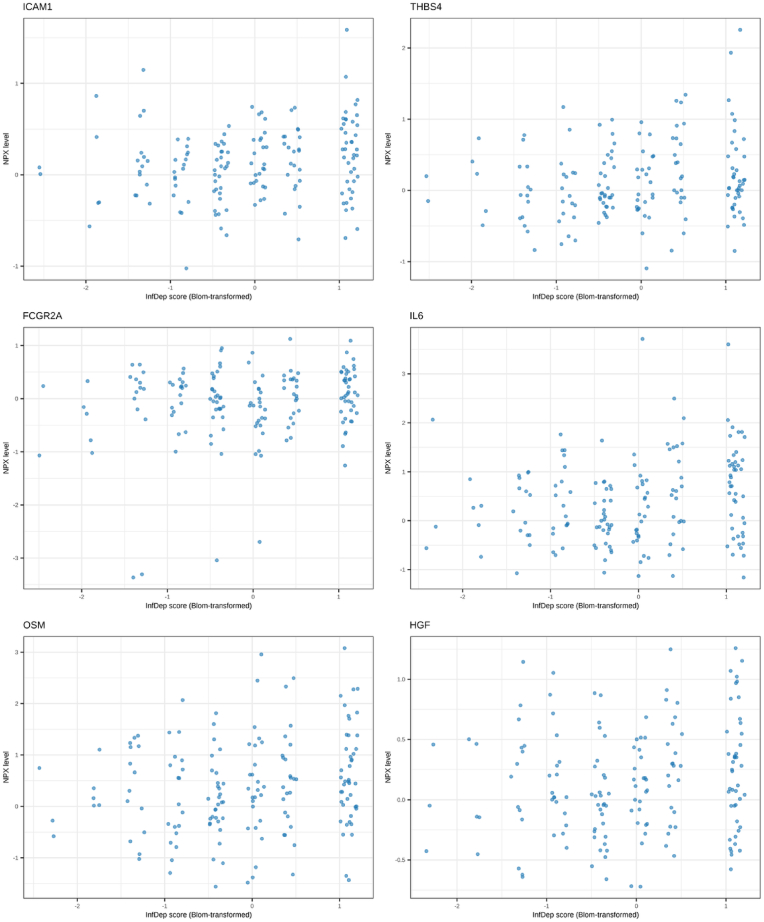
Scatterplots of protein levels (measured with proximity extension assay) of proteins that were most robustly associated with increased depressive symptoms tentatively caused by inflammation (InfDep score). The InfDep scores (0-9) were transformed using the Blom method to achieve an approximately normal distribution. Coefficients of the linear regressions adjusted for age and sex with the corresponding FDR-corrected P-values were: 0.08 (P = 0.050) for intercellular adhesion molecule 1 (ICAM1), 0.10 (P = 0.050) for thrombospondin-4 (THBS4), 0.13 (P = 0.050) for low affinity immunoglobulin gamma Fc region receptor II-a (FCGR2A), 0.19 (P = 0.031) for interleukin-6 (IL6), 0.17 (P = 0.037) for oncostatin M (OSM) and 0.08 (P = 0.037) for hepatocyte growth factor (HGF).

### 3.3 Specificity to inflammatory depressive symptoms and internal consistency

To examine whether the proteins most strongly associated with the InfDep score were also associated to the remaining PHQ-9 symptoms, we did linear regressions using the sum of items 1, 2, 6, 7, and 9 as the outcome, applying the same Blom transformation and adjusting for age and sex as in the InfDep analyses.

With the exception of IL-6, none of these markers showed significant associations with the remaining PHQ-9 items that were not classified as inflammatory depressive symptoms (supplementary material 3).

We also investigated the internal consistency of the InfDep score by calculating Cronbach's alpha (using R-package psych, https://personality-project.org/r/psych/v2.5.6). The alpha for all patients was 0.47, 95 % CI [0.32, 0.60], and differed slightly between studies where Omega-3 study participants had lower alpha of 0.38, 95 % CI [0.14, 0.57] compared to in the Probiotics study where alpha was 0.57, 95 % CI [0.37, 0.72].

## 4 Discussion

In this cohort, we found evidence for an association between inflammation and a depression profile characterized by more severe fatigue, sleep and appetite disturbances. Plasma proteins that were most robustly associated with this inflammatory depression symptom profile – significant across models using both the dichotomized and continuous InfDep scores – were IL-6, OSM, HGF, ICAM1, THBS4 and FCGR2A. Although the effect sizes were quite modest, higher levels of all these biomarkers were related to more severe inflammatory depressive symptoms. These findings are consistent with previous reports linking inflammation to specific depressive symptoms but extend the evidence by applying a broader proteomic approach that includes a larger panel of biomarkers.

### 4.1 Possible roles of main findings in depression pathophysiology

As shown in network analyses, five of the six above-mentioned proteins were highly functionally interconnected and related to established inflammatory biological processes, even though not all of them have been specifically studied in relation to MDD. We found that IL-6 was significantly associated with severity of inflammatory depressive symptoms and, to a lesser extent, with other depressive symptoms. IL-6 is a canonical pro-inflammatory cytokine, and numerous studies have reported elevated levels in MDD, especially in those with an inflammatory depressive symptom profile (Frank et al., 2021). Monoclonal antibodies treatments targeting IL-6 signalling are currently being used for autoimmune disorders, and such treatments may also have antidepressant effects (Backhaus et al., 2015). Our results add to the accumulating evidence linking IL-6 to inflammatory depressive symptoms, indicating it might be a key biomarker for “inflammatory depression”. Another pro-inflammatory member of the IL-6 cytokine family is OSM, secreted from activated immune cells such as macrophages. Elevated plasma and cerebrospinal fluid OSM levels have been reported in MDD patients compared to controls (Franzen et al., 2020; Lindahl et al., 2025; Oh et al., 2025). Previous studies, together with the findings from the present study, suggest that OSM might play an important role in pathophysiological processes underlying inflammatory depressive symptoms. HGF is considered a neuroprotective agent with anti-inflammatory and angiogenic properties in the central nervous system (Desole et al., 2021). It therefore seems paradoxical that we (Lindahl et al., 2025) and others (Oh et al., 2025) have previously found elevated HGF levels in depressed patients compared to controls, and our results suggest that HGF might be increased in patients with more severe inflammatory depressive symptoms. Possibly, HGF elevation is a regulatory mechanism where this protein modulates inflammation and immune response in the brain (Desole et al., 2021). Adhesion molecule ICAM1 was also related to inflammatory depressive symptoms. It is hypothesized to be a possible marker for blood-brain-barrier dysfunction, enabling neuroinflammation (Müller, 2019). Previous studies have shown elevated ICAM1 levels in psychiatric conditions such as MDD, bipolar disorder and schizophrenia, irrespective of symptom profile (Müller, 2019), suggesting that this biomarker may not be specific to inflammatory depressive symptoms but rather reflect general psychiatric symptom burden.

THBS4 was also elevated in patients with higher InfDep scores. THBS4 increases during pathological insults and has been shown to induce peripheral inflammatory activity in macrophages, but in the brain increased levels have been found to induce synaptogenesis through unknown mechanisms (Genaro and Luo, 2024). In psychiatric disorders, THBS4 has been proposed as a potential mediator of the impaired hippocampal synaptic pruning hypothesized to contribute to the development of schizophrenia (Cocchi et al., 2016). Lastly, FCGR2A was significantly elevated in patients with higher InfDep scores. As a receptor for the Fc portion of immunoglobulins and pentraxins such as CRP, it has integral functions for both the adaptive and the innate immune system (Lu et al., 2018). To the best of our knowledge, this is the first time THBS4 and FCGR2A have been investigated in MDD, and their roles in the pathophysiology of inflammatory depressive symptoms needs to be further elucidated.

### 4.2 Comparison with previous studies on inflammatory depressive symptoms

We have only found two previous studies that used proteomics to investigate inflammatory biomarkers in relation to depressive symptoms tentatively linked to inflammation. Both studies were conducted on the Netherlands study of Depression and Anxiety (NESDA) cohort and used the same proteomic analytes on different subsets (Lamers et al., 2016; van Haeringen et al., 2023). There was almost no overlap between the proteins analyzed in ours and these two studies. To assess whether our findings are consistent with previous results, we performed an additional STRING network analysis (supplementary material 2) on IL-6, HGF, OSM, THBS4, FCGR2A and ICAM1 and the proteins which were linked to inflammatory depressive symptoms in the two NESDA studies. This analysis showed large interaction scores between many proteins, indicating that these proteins are highly inter-connected in physiological processes which might cause inflammatory depressive symptoms.

In the present study, higher BMI – as a surrogate marker for obesity and overweight – was considered an integral part of the inflammatory depression endophenotype and we did therefore not adjust for this variable in our analyses, similarly to previous publications (Lamers et al., 2016; van Haeringen et al., 2023). Overweight has been suggested as a key factor underlying the inflammatory depressive phenotype with a bidirectional relationship – both causing inflammation and being the result of depressive symptoms (Lasserre et al., 2014; Luppino et al., 2010; Milaneschi et al., 2019). Future studies aimed at clarifying the causality between overweight, obesity and inflammatory depressive symptoms should examine whether targeted weight loss leads to improvement of these symptoms parallelled by a normalization of inflammatory markers.

### 4.3 Strengths and limitations

Strengths of the study include the investigation of a comprehensive panel of proteins tentatively connected to inflammatory depressive symptoms in a clinically well-defined MDD sample and using robust and conservative statistical methods that limit the risk of over-fitting, *e.g.* by using cross-validation in the PLS/PLS-DA analyses and using FDR-corrected linear regressions for our main conclusions. This study, however, has several limitations. Being a cross-sectional study we cannot draw conclusions on causality. Furthermore, we relied solely on plasma protein markers, and conducting analogous analyses in cerebrospinal fluid would be a particularly informative next step to relate peripheral biomarkers to corresponding central processes. Furthermore, the InfDep score which we extracted from the PHQ-9 self-rating scale can be criticized. Internal consistency among the InfDep items was low and varied slightly between cohorts, which is not unexpected given that participants in the probiotics study had higher InfDep scores overall, possibly due to enrichment for low-grade inflammation. The overall low alpha might be caused by the small number of items which constitute the InfDep score, or that multiple factors influence these (Tavakol and Dennick, 2011). It is therefore plausible that a more precise rating scale targeting specific depressive symptoms would have been better suited to detect weaker associations than was possible in this study, reducing the risk of false-negative findings associated with our approach. We recognize that the PHQ-9 items included in the InfDep score should not be interpreted as specifically reflecting an atypical depressive phenotype since they assess both increased and decreased appetite and sleep disturbances – they were, however, pre-selected as a crude measure of inflammatory depressive symptoms based on the results from a previous meta-analysis linking these specific items with increased CRP levels in MDD patients (Jokela et al., 2016). Future studies linking inflammatory biomarkers to specific depressive symptoms using comprehensive rating scales could specify and possibly expand the concept of “inflammatory depressive symptoms”. Lastly, out of the markers most robustly associated with InfDep, only IL-6 showed significant associations with the remaining PHQ-9 items, albeit to a lower degree than for the items which constitute the InfDep score. We interpret this finding cautiously since IL-6 has been repeatedly linked to an inflammatory symptom profile (Frank et al., 2021; Fried et al., 2020). Overall, our results suggest a degree of specificity between immunometabolic alterations and inflammatory depressive symptoms, rather than a generalized relationship with overall depression severity.

### 4.4 Concluding remarks

In conclusion, we here identified several plasma protein biomarkers of potential relevance for a subset of depressive symptoms that align with the concept of “inflammatory depression”. If these results are replicated across studies, we suggest that they may provide valuable insights to the pathophysiological mechanisms underlying inflammatory depression and methods for treating this condition.

## Funding

This study was funded by the 10.13039/501100004359Swedish Research Council (grant number 2020-01428), Swedish governmental funding of clinical research (ALF), the OM Persson Foundation, the Ellen and Henrik Sjöbring Foundation, the Söderderström Königska Foundation, the Lions Research Foundation, the 10.13039/501100007687Swedish Society of Medicine, Skåne University Hospital Donations, the Per-Eric and Ulla Schybergs Foundation, the Professor Bror Gadelius memorial fund, grants from the province of Scania, the Dr P Håkansson Foundation, the 10.13039/501100016049Thure Carlsson Foundation, and 10.13039/501100013896BioGaia AB.

## CRediT authorship contribution statement

**Filip Ängeby:** Conceptualization, Data curation, Formal analysis, Investigation, Methodology, Software, Validation, Visualization, Writing – original draft. **Filip Ventorp:** Conceptualization, Data curation, Methodology, Software, Supervision, Writing – review & editing. **Miranda Stiernborg:** Formal analysis, Methodology, Software, Writing – review & editing. **Klara Suneson:** Data curation, Investigation, Writing – review & editing. **Gustav Söderberg Veibäck:** Data curation, Investigation, Writing – review & editing. **Jesper Lindahl:** Data curation, Investigation, Writing – review & editing. **Johanna Tjernberg:** Data curation, Investigation, Project administration, Writing – review & editing. **Darya Ståhl:** Data curation, Investigation, Project administration, Writing – review & editing. **Jonas Berge:** Methodology, Supervision, Writing – review & editing. **Catharina Lavebratt:** Conceptualization, Methodology, Supervision, Writing – review & editing. **Daniel Lindqvist:** Conceptualization, Funding acquisition, Investigation, Methodology, Project administration, Supervision, Writing – original draft.

## Declaration of competing interest

The authors declare that they have no known competing financial interests or personal relationships that could have appeared to influence the work reported in this paper.

## Data Availability

The authors do not have permission to share data.

## Footnotes

Appendix A Supplementary data to this article can be found online at https://doi.org/10.1016/j.bbih.2026.101311.
