# Supplementary material for: Plasma proteomic profile of inflammatory depressive symptoms
Source: Brain Behav Immun Health. 2026 Jul 23;56:101311. doi: 10.1016/j.bbih.2026.101311 (PMC13446103; doi:10.1016/j.bbih.2026.101311)
Supplement: Supplementary file 1 [file mmc2.docx]

**Supplementary material 2**

STRING network analysis of all proteins which were significant in our study, Lamers et al. 2016 and van Haeringen et al. 2023 except for C-peptide which was not found in the STRING database. Each line indicates a “confidence score” which in this case was set to > 0.15 (low confidence). Abbreviations of all proteins and a summary of their function (provided by STRING) is listed in the table below.

| ADIPOQ | Adiponectin; Important adipokine involved in the control of fat metabolism and insulin sensitivity, with direct anti-diabetic, anti-atherogenic and anti-inflammatory activities. Stimulates AMPK phosphorylation and activation in the liver and the skeletal muscle, enhancing glucose utilization and fatty-acid combustion. Antagonizes TNF-alpha by negatively regulating its expression in various tissues such as liver and macrophages, and also by counteracting its effects. Inhibits endothelial NF-kappa-B signaling through a cAMP-dependent pathway. May play a role in cell growth, angiogenesis [...] |
| --- | --- |
| AHSG | Alpha-2-HS-glycoprotein chain A; Promotes endocytosis, possesses opsonic properties and influences the mineral phase of bone. Shows affinity for calcium and barium ions; Belongs to the fetuin family. |
| AMBP | Inter-alpha-trypsin inhibitor light chain; Inter-alpha-trypsin inhibitor inhibits trypsin, plasmin, and lysosomal granulocytic elastase. Inhibits calcium oxalate crystallization; In the N-terminal section; belongs to the calycin superfamily. Lipocalin family. |
| ANGPT2 | Angiopoietin-2; Binds to TEK/TIE2, competing for the ANGPT1 binding site, and modulating ANGPT1 signaling. Can induce tyrosine phosphorylation of TEK/TIE2 in the absence of ANGPT1. In the absence of angiogenic inducers, such as VEGF, ANGPT2-mediated loosening of cell-matrix contacts may induce endothelial cell apoptosis with consequent vascular regression. In concert with VEGF, it may facilitate endothelial cell migration and proliferation, thus serving as a permissive angiogenic signal. |
| APCS | Serum amyloid P-component(1-203); Can interact with DNA and histones and may scavenge nuclear material released from damaged circulating cells. May also function as a calcium-dependent lectin; Belongs to the pentraxin family. |
| APOB | Apolipoprotein B-100; Apolipoprotein B is a major protein constituent of chylomicrons (apo B-48), LDL (apo B-100) and VLDL (apo B-100). Apo B- 100 functions as a recognition signal for the cellular binding and internalization of LDL particles by the apoB/E receptor. |
| B2M | Beta-2-microglobulin form pI 5.3; Component of the class I major histocompatibility complex (MHC). Involved in the presentation of peptide antigens to the immune system. Exogenously applied M.tuberculosis EsxA or EsxA-EsxB (or EsxA expressed in host) binds B2M and decreases its export to the cell surface (total protein levels do not change), probably leading to defects in class I antigen presentation. Belongs to the beta-2-microglobulin family. |
| C3 | Complement C3c alpha' chain fragment 1; C3 plays a central role in the activation of the complement system. Its processing by C3 convertase is the central reaction in both classical and alternative complement pathways. After activation C3b can bind covalently, via its reactive thioester, to cell surface carbohydrates or immune aggregates. [C3-beta-c]: Acts as a chemoattractant for neutrophils in chronic inflammation. |
| CRP | C-reactive protein(1-205); Displays several functions associated with host defense: it promotes agglutination, bacterial capsular swelling, phagocytosis and complement fixation through its calcium-dependent binding to phosphorylcholine. Can interact with DNA and histones and may scavenge nuclear material released from damaged circulating cells. |
| CTSD | Cathepsin D heavy chain; Acid protease active in intracellular protein breakdown. Plays a role in APP processing following cleavage and activation by ADAM30 which leads to APP degradation. Involved in the pathogenesis of several diseases such as breast cancer and possibly Alzheimer disease. |
| FABP4 | Fatty acid-binding protein, adipocyte; Lipid transport protein in adipocytes. Binds both long chain fatty acids and retinoic acid. Delivers long-chain fatty acids and retinoic acid to their cognate receptors in the nucleus. Belongs to the calycin superfamily. Fatty-acid binding protein (FABP) family. |
| FCGR2A | Low affinity immunoglobulin gamma Fc region receptor II-a; Binds to the Fc region of immunoglobulins gamma. Low affinity receptor. By binding to IgG it initiates cellular responses against pathogens and soluble antigens. Promotes phagocytosis of opsonized antigens. |
| GSTA5 | Glutathione S-transferase alpha 5. |
| HGF | Hepatocyte growth factor alpha chain; Potent mitogen for mature parenchymal hepatocyte cells, seems to be a hepatotrophic factor, and acts as a growth factor for a broad spectrum of tissues and cell types. Activating ligand for the receptor tyrosine kinase MET by binding to it and promoting its dimerization. Belongs to the peptidase S1 family. Plasminogen subfamily. |
| HP | Haptoglobin alpha chain; As a result of hemolysis, hemoglobin is found to accumulate in the kidney and is secreted in the urine. Haptoglobin captures, and combines with free plasma hemoglobin to allow hepatic recycling of heme iron and to prevent kidney damage. Haptoglobin also acts as an antioxidant, has antibacterial activity, and plays a role in modulating many aspects of the acute phase response. Hemoglobin/haptoglobin complexes are rapidly cleared by the macrophage CD163 scavenger receptor expressed on the surface of liver Kupfer cells through an endocytic lysosomal degradation pathway. |
| ICAM1 | Intercellular adhesion molecule 1; ICAM proteins are ligands for the leukocyte adhesion protein LFA-1 (integrin alpha-L/beta-2). During leukocyte trans-endothelial migration, ICAM1 engagement promotes the assembly of endothelial apical cups through ARHGEF26/SGEF and RHOG activation. (Microbial infection) Acts as a receptor for Coxsackievirus A21 capsid proteins. |
| IGFBP1 | Insulin-like growth factor-binding protein 1; IGF-binding proteins prolong the half-life of the IGFs and have been shown to either inhibit or stimulate the growth promoting effects of the IGFs on cell culture. They alter the interaction of IGFs with their cell surface receptors. Promotes cell migration. |
| IGFBP2 | Insulin-like growth factor-binding protein 2; Inhibits IGF-mediated growth and developmental rates. IGF- binding proteins prolong the half-life of the IGFs and have been shown to either inhibit or stimulate the growth promoting effects of the IGFs on cell culture. They alter the interaction of IGFs with their cell surface receptors. |
| IL6 | Interleukin-6; Cytokine with a wide variety of biological functions. It is a potent inducer of the acute phase response. Plays an essential role in the final differentiation of B-cells into Ig-secreting cells Involved in lymphocyte and monocyte differentiation. Acts on B-cells, T-cells, hepatocytes, hematopoietic progenitor cells and cells of the CNS. Required for the generation of T(H)17 cells. Also acts as a myokine. It is discharged into the bloodstream after muscle contraction and acts to increase the breakdown of fats and to improve insulin resistance. It induces myeloma and plasm [...] |
| INS | Insulin A chain; Insulin decreases blood glucose concentration. It increases cell permeability to monosaccharides, amino acids and fatty acids. It accelerates glycolysis, the pentose phosphate cycle, and glycogen synthesis in liver. |
| LEP | Leptin; Key player in the regulation of energy balance and body weight control. Once released into the circulation, has central and peripheral effects by binding LEPR, found in many tissues, which results in the activation of several major signaling pathways. In the hypothalamus, acts as an appetite-regulating factor that induces a decrease in food intake and an increase in energy consumption by inducing anorexinogenic factors and suppressing orexigenic neuropeptides, also regulates bone mass and secretion of hypothalamo- pituitary-adrenal hormones. In the periphery, increases basal me [...] |
| MIF | Macrophage migration inhibitory factor; Pro-inflammatory cytokine. Involved in the innate immune response to bacterial pathogens. The expression of MIF at sites of inflammation suggests a role as mediator in regulating the function of macrophages in host defense. Counteracts the anti-inflammatory activity of glucocorticoids. Has phenylpyruvate tautomerase and dopachrome tautomerase activity (in vitro), but the physiological substrate is not known. It is not clear whether the tautomerase activity has any physiological relevance, and whether it is important for cytokine activity. |
| MSLN | Megakaryocyte-potentiating factor; Membrane-anchored forms may play a role in cellular adhesion; Belongs to the mesothelin family. |
| OSM | Oncostatin-M; Growth regulator. Inhibits the proliferation of a number of tumor cell lines. Stimulates proliferation of AIDS-KS cells. It regulates cytokine production, including IL-6, G-CSF and GM-CSF from endothelial cells. Uses both type I OSM receptor (heterodimers composed of LIFR and IL6ST) and type II OSM receptor (heterodimers composed of OSMR and IL6ST). Involved in the maturation of fetal hepatocytes, thereby promoting liver development and regeneration (By similarity). Belongs to the LIF/OSM family. |
| PLAT | Tissue-type plasminogen activator chain A; Converts the abundant, but inactive, zymogen plasminogen to plasmin by hydrolyzing a single Arg-Val bond in plasminogen. By controlling plasmin-mediated proteolysis, it plays an important role in tissue remodeling and degradation, in cell migration and many other physiopathological events. Plays a direct role in facilitating neuronal migration; Belongs to the peptidase S1 family. |
| PLAU | Urokinase-type plasminogen activator short chain A; Specifically cleaves the zymogen plasminogen to form the active enzyme plasmin. |
| PLGRKT | Plasminogen receptor (KT); Receptor for plasminogen. Regulates urokinase plasminogen activator-dependent and stimulates tissue-type plasminogen activator- dependent cell surface plasminogen activation. Proposed to be part of a local catecholaminergic cell plasminogen activation system that regulates neuroendocrine prohormone processing. Involved in regulation of inflammatory response; regulates monocyte chemotactic migration and matrix metalloproteinase activation, such as of MMP2 and MMP9. |
| PROS1 | Vitamin K-dependent protein S; Anticoagulant plasma protein; it is a cofactor to activated protein C in the degradation of coagulation factors Va and VIIIa. It helps to prevent coagulation and stimulating fibrinolysis. |
| PRSS8 | Prostasin heavy chain; Possesses a trypsin-like cleavage specificity with a preference for poly-basic substrates. Stimulates epithelial sodium channel (ENaC) activity through activating cleavage of the gamma subunits (SCNN1G). |
| PSAT1 | Phosphoserine aminotransferase; Catalyzes the reversible conversion of 3- phosphohydroxypyruvate to phosphoserine and of 3-hydroxy-2-oxo-4- phosphonooxybutanoate to phosphohydroxythreonine. Belongs to the class-V pyridoxal-phosphate-dependent aminotransferase family. SerC subfamily. |
| SERPINA3 | Alpha-1-antichymotrypsin His-Pro-less; Although its physiological function is unclear, it can inhibit neutrophil cathepsin G and mast cell chymase, both of which can convert angiotensin-1 to the active angiotensin-2. |
| SHBG | Sex hormone-binding globulin; Functions as an androgen transport protein, but may also be involved in receptor mediated processes. Each dimer binds one molecule of steroid. Specific for 5-alpha-dihydrotestosterone, testosterone, and 17-beta-estradiol. Regulates the plasma metabolic clearance rate of steroid hormones by controlling their plasma concentration. |
| THBS4 | Thrombospondin-4; Adhesive glycoprotein that mediates cell-to-cell and cell-to- matrix interactions and is involved in various processes including cellular proliferation, migration, adhesion and attachment, inflammatory response to CNS injury, regulation of vascular inflammation and adaptive responses of the heart to pressure overload and in myocardial function and remodeling. Binds to structural extracellular matrix (ECM) proteins and modulates the ECM in response to tissue damage, contributing to cardioprotective and adaptive ECM remodeling. Plays a role in ER stress response, via it [...] |
| VTN | Vitronectin V10 subunit; Vitronectin is a cell adhesion and spreading factor found in serum and tissues. Vitronectin interact with glycosaminoglycans and proteoglycans. Is recognized by certain members of the integrin family and serves as a cell-to-substrate adhesion molecule. Inhibitor of the membrane-damaging effect of the terminal cytolytic complement pathway. |
| VWF | Von Willebrand antigen 2; Important in the maintenance of hemostasis, it promotes adhesion of platelets to the sites of vascular injury by forming a molecular bridge between sub-endothelial collagen matrix and platelet- surface receptor complex GPIb-IX-V. Also acts as a chaperone for coagulation factor VIII, delivering it to the site of injury, stabilizing its heterodimeric structure and protecting it from premature clearance from plasma. |
