# Supplementary material for: Plasma proteomic profile of inflammatory depressive symptoms
Source: Brain Behav Immun Health. 2026 Jul 23;56:101311. doi: 10.1016/j.bbih.2026.101311 (PMC13446103; doi:10.1016/j.bbih.2026.101311)
Supplement: Supplementary file 2 [file mmc3.docx]

Supplementary material 3

| ***Protein marker*** | ***Protein panel*** | ***Regression coefficient*** | ***Non-adjusted P-value*** | ***FDR-corrected P-value*** |
| --- | --- | --- | --- | --- |
| IL6 | Inflammation | 0.18 | 0.012 | 0.031 |
| HGF | Inflammation | 0.02 | 0.558 | 0.559 |
| OSM | Inflammation | 0.13 | 0.069 | 0.103 |
| THBS4 | Cardiometabolic | 0.05 | 0.290 | 0.867 |
| FCGR2A | Cardiometabolic | 0.02 | 0.730 | 1.000 |
| ICAM1 | Cardiometabolic | -0.01 | 0.830 | 1.000 |

We performed a linear regression on the sum of PHQ-9 items which did not constitute the Inflammatory Depressive Symptom Score (InfDep score, items # 1, 2, 6, 7, 8 and 9) and the proteins that were most robustly associated with InfDep scores, to test our assumption that specific depressive symptoms are associated with inflammation (as indicated by previous studies referred to in the main article). The sum of the non-InfDep PHQ-9 items were Blom-transformed prior to regression, which was otherwise performed in the same way as for the InfDep score items adjusting for sex and age. As the table above indicates, only IL-6 showed a significant association with non-InfDep PHQ-9 items both before and after FDR-correction.
